# CADM1 is expressed as multiple alternatively spliced functional and dysfunctional isoforms in human mast cells

**DOI:** 10.1016/j.molimm.2012.08.024

**Published:** 2013-04

**Authors:** Elena P. Moiseeva, Mark L. Leyland, Peter Bradding

**Affiliations:** a Institute for Lung Health, Dept. of Infection, Immunity and Inflammation, University of Leicester, Leicester, UK; b Department of Biochemistry, University of Leicester, Leicester, UK

**Keywords:** ASMCs, airway smooth muscle cells, CADM1, cell adhesion molecule 1, ECD, extracellular domain, HLMCs, human lung mast cells, LFs, lung fibroblasts, MCs, mast cells, PMA, phorbol-12-myristate-13-acetate, SP, alternatively spliced isoform, TSLC1, tumour suppressor of lung cancer 1, Human, Mast cells, Cell adhesion molecule 1, Alternative splicing, Cell adhesion

## Abstract

Cell adhesion molecule 1 (CADM1) is implicated in the pathogenesis of several diseases and is responsible for adhesion and survival of mast cells (MCs). Differential expression of CADM1 isoforms was found in different species. We previously cloned SP4, SP1, SP6 and a dysfunctional isoform from human lung MCs (HLMCs) and the MC line HMC-1. The aim of this study was to identify all isoforms expressed in human MCs. The functional isoforms SP4, SP1, SP6 and SP3, with alternative splicing between exons 7/11, were detected in human MCs by RT-PCR. Two dysfunctional isoforms with alternative splicing of cryptic exons A and B between exons 1/2, leading to premature termination of translation, were found in ∼40% of MC specimens. Sequencing of genomic DNA showed that splicing of cryptic exon B did not result from specific SNPs within this exon or its putative splice branch point. Highly glycosylated CADM1 (∼105 kDa) was detected by western blotting, but an extracellular domain (∼95 kDa) was found only in the culture medium from HLMCs, but not HMC-1 cells, indicating differential protein expression. Transfection of SP1 and SP6, but not SP4, reduced adhesion of HMC-1 cells to human lung fibroblasts but not airway smooth muscle cells. Hence, dysfunctional and functional CADM1 isoforms are found in human MCs. The longer SP1 and SP6 were most evident in differentiated HLMCs and displayed differential adhesion compared to SP4. These multiple isoforms are likely to contribute to MC function in both health and disease.

## Graphical abstract

**Graphical abstract:**

**Figure:**
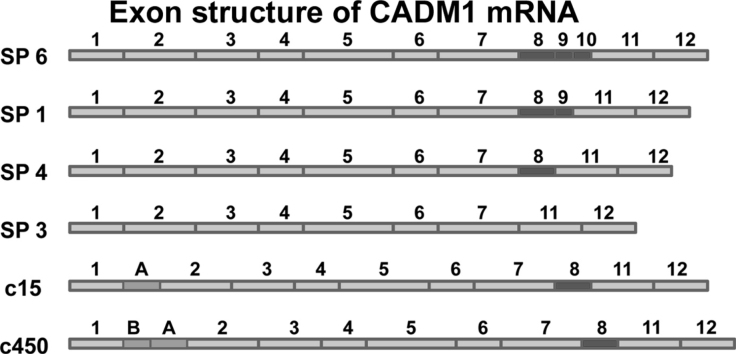

**Highlights:**

► Human mast cells express 6 alternatively spliced CADM1 isoforms: 4 functional and 2 dysfunctional. ► Long CADM1 SP1 and SP6 are restricted to differentiated human lung mast cells. ► Expression of mixed isoforms reduces adhesion of human mast cells to lung fibroblasts. ► Dysfunctional CADM1 isoforms are found in 40% of mast cells. ► Dysfunctional isoforms limit CADM1 expression and may predispose to various diseases.

## 1 Introduction

The *CADM1* (cell adhesion molecule 1) gene encodes a cell adhesion receptor CADM1 (also known as IGSF4, TSLC1, Necl-2, SynCAM1), which belongs to an immunoglobulin family of ten genes, which also include *CADM2-4*, *PVRL1-4*, *PVR*, and *CRTAM*, according to Ensembl (Flicek et al., 2011). Human CADM1 was originally identified as a tumour suppressor of lung cancer 1 (TSLC1; SP4) (Kuramochi et al., 2001). It is essential for human health, since modulation of expression, SNPs or mutations in CADM1 have been implicated in several diseases including cancer, autism spectrum disorder and venous thrombosis (Gomyo et al., 1999; Hasstedt et al., 2009; Zhiling et al., 2008). CADM1 expression is significantly reduced in neuroblastoma and cancers of the lung, oesophagus and skin; furthermore, it is inversely correlated with cancer progression (Ito et al., 2003b; Nowacki et al., 2008; Uchino et al., 2003; You et al., 2010). CADM1 expression is also reduced in endothelial cells in venous thrombosis (Hasstedt et al., 2009). In contrast, CADM1 is upregulated in adult T-cell leukaemia/lymphoma and acute T-cell leukaemia, facilitating tissue infiltration (Dewan et al., 2008; Nakahata et al., 2012; Sasaki et al., 2005). CADM1 expression is also increased in the neuro-developmental disorder Rett syndrome (Nectoux et al., 2010).

CADM1 is involved in adhesion via homophilic and heterophilic interactions with counter-receptors on other cells. All identified counter-receptors for CADM1 belong to the same family. They include CADM1 itself, CADM2, CADM3, nectin3 and CRTAM (Fogel et al., 2007; Galibert et al., 2005; Masuda et al., 2002; Shingai et al., 2003). CD155 binding to CADM1 is controversial (Shingai et al., 2003; Wakayama et al., 2007).

CADM1 is expressed by mast cells (MCs) and supports MC adhesion to human airway smooth muscle cells (ASMCs), mouse fibroblasts and nerves (Furuno et al., 2005; Ito et al., 2003a; Yang et al., 2006). We have shown recently that CADM1 is expressed as several isoforms (illustrated in Fig. 2 below) in human MCs (Moiseeva et al., 2012). CADM1 downregulation in human lung MCs (HLMCs) and the immature human MC line HMC-1 decreases their survival and increases caspase-3/7 activation compared to control cells (Moiseeva et al., 2012). Moreover, we have shown that overexpression of the SP4 and SP1 isoforms, which differ only by 11 amino acid residues encoded by exon 9, differentially modulates homotypic MC adhesion and survival (Moiseeva et al., 2012). Consequently, CADM1 plays a key role in MC biology.

The CADM1 mRNA undergoes alternative splicing between exons 7 and 11 of the *CADM1* gene in several species (Biederer, 2006; Flicek et al., 2011; Sayers et al., 2011). The alternatively spliced isoforms SP4 (exons 1–8/11–12) and SP3 (exons 1–7/11–12), named here as by Biederer (2006), are the most common and listed in the NCBI gene database (Sayers et al., 2011). In addition, cDNAs for human SP5 (exon 1–intron 7), SP2 (exons 1–7/9–12) and SP1 (exons 1–9/11–12) are present in DNA databases. Nonetheless, there is little information on expression of different CADM1 isoforms in humans despite its significance for human health. We have cloned novel SP6 (exons 1–12) and dysfunctional c15 (exon 1/A/2–8/11–12) from human MCs (Moiseeva et al., 2012). Moreover, several other isoforms, including soluble SP5 (exon 1–intron 7) and CADM1b (exons 1–7/9/11–12) have been found in mouse MCs and brain (Hagiyama et al., 2011; Koma et al., 2004). CADM1 is a protein with a variable protein core of 46 kDa (SP3)–52 kDa (SP6) and diverse glycosylation (∼50 kDa). N-linked glycosylation (∼25 kDa) is mapped to Ig domains (Chen et al., 2009; Liu et al., 2005; Wollscheid et al., 2009), whereas the site for O-linked glycosylation (∼25 kDa) is not known. The NetOGlyc 3.1 server (Julenius et al., 2005) locates the O-glycosylation sites in CADM1 to 17 threonines encoded by exon 8 and 4 threonines encoded by exon 9. The shortest SP3 isoform corresponds to a glycosylated CADM1 of ∼70 kDa (Hagiyama et al., 2011) and is, therefore, likely to be only N-glycosylated, as it lacks sequences encoded by exons 8 and 9. SP4 is ∼105 kDa and is both N- and O-glycosylated (Ito et al., 2003a). The longer isoforms SP1 and SP6 are expected to exhibit maximal O-glycosylation due to the presence of exons 8 and 9. Consistent with this, Hagiyama et al. (2011) have demonstrated that inclusion of exon 9 in SP3 to produce CADM1b increases protein weight by ∼5 kDa. We also have found that both SP4 and SP1 overexpressed in HMC-1 cells are about 105 kDa, but SP1 has slightly reduced mobility in SDS-PAGE compared to SP4 (Moiseeva et al., 2012).

There are significant differences in expression of CADM1 isoforms in murine and human MCs. Murine MCs express SP4 and soluble SP5 (Ito et al., 2003a; Koma et al., 2004); the latter is considered to reduce homophilic cell adhesion, mediated by CADM1. We have cloned a cryptic (c15) and three functional (SP4, SP1 and SP6) isoforms from HLMCs and HMC-1 cells (Moiseeva et al., 2012). The non-differentiated HMC-1 cells express only the functional SP4 isoform, which promotes homotypic MC adhesion and survival (Moiseeva et al., 2012), consistent with the neoplastic nature of these cells. In contrast, differentiated HLMCs express SP4 and longer isoforms SP1 and SP6; SP1 reduces survival and homotypic MC adhesion (Moiseeva et al., 2012). Since CADM1 isoforms have different functions in MCs and there are marked differences in expression of CADM1 isoforms in human and mouse MCs, the aim of this study was (i) to identify all CADM1 isoforms expressed in differentiated human HLMCs and the neoplastic MC lines HMC-1 and LAD2, which were derived from patients with MC leukaemia/sarcoma (Butterfield et al., 1988; Kirshenbaum et al., 2003) and (ii) investigate the roles of HLMC-specific isoforms in MC adhesion. Here we have identified an additional SP3 and another novel dysfunctional isoform c450 in human MCs, but not SP5, in addition to previously cloned isoforms (Moiseeva et al., 2012). We also show that expression of mixed isoforms in HMC-1 cells results in reduced adhesion to lung fibroblasts.

## 2 Materials and methods

### 2.1 Cell culture

The human MC line HMC-1, obtained from Dr Butterfield (Butterfield et al., 1988), was cultured in IMDM with 10% FCS as described previously (Hollins et al., 2008). HLMCs were obtained from healthy lung acquired at surgery for carcinoma using anti-CD117-coated Dynabeads (Sanmugalingam et al., 2000). The final purity of HLMCs used in experiments was >99%. HLMCs were cultured in DMEM supplemented with 10% FCS and cytokines (100 ng/ml SCF, 50 ng/ml IL-6, and 10 ng/ml IL-10) (Moiseeva et al., 2012). The study was approved by the Leicestershire Research Ethics Committee and all participants gave written informed consent.

ASMCs were isolated using explant culture of ASM bundles as previously described (Kaur et al., 2006) and expressed smooth muscle-specific antigens α-smooth muscle actin and smooth muscle myosin with efficiency 93% and 60%, respectively. All patients gave written informed consent and the study was approved by the Leicestershire, Northamptonshire and Rutland Research Ethics Committee. ASMCs were cultured in DMEM supplemented with 10% FBS, antibiotic/antimycotic agents and non-essential amino acids (Kaur et al., 2006). Parenchymal LFs were isolated using explant culture from healthy areas of lung tissue obtained at surgery for carcinoma as previously described (Pechkovsky et al., 2010). All patients gave written informed consent and the study was approved by the Leicestershire, Northamptonshire and Rutland Research Ethics Committee. LFs were grown in the same conditions as ASMCs, and expressed fibroblast-specific antigens 1B10 and Thy-1 with efficiency 97%. ASMCs and LFs were used at passages 3–5.

### 2.2 CADM1 cDNA amplification and isoform analysis

Total RNA, isolated previously (Moiseeva et al., 2012) from the cell lines, HMC-1 and LAD2, and HLMC specimens, D450 and D449, was used in this study. CADM1 cDNA was synthesised using AccuScript RT-PCR kit (Stratagene) in nested RT-PCR with primers F1–R1 (all oligonucleotide primers are shown in Table 1), followed by primers F2–R2. For SP5, intron7R primer [identical to R3 in (Koma et al., 2004)] was used for reverse transcription with AccuScript RT-PCR kit (Stratagene), PCR was performed using primers exon6F and intron7R to amplify a cDNA fragment. For full-length SP5 cDNA, nested RT-PCR was performed with primers F1–intron7R, followed by primers F2–intron7R or F2–ExIn7R.

**Fig. 1:**
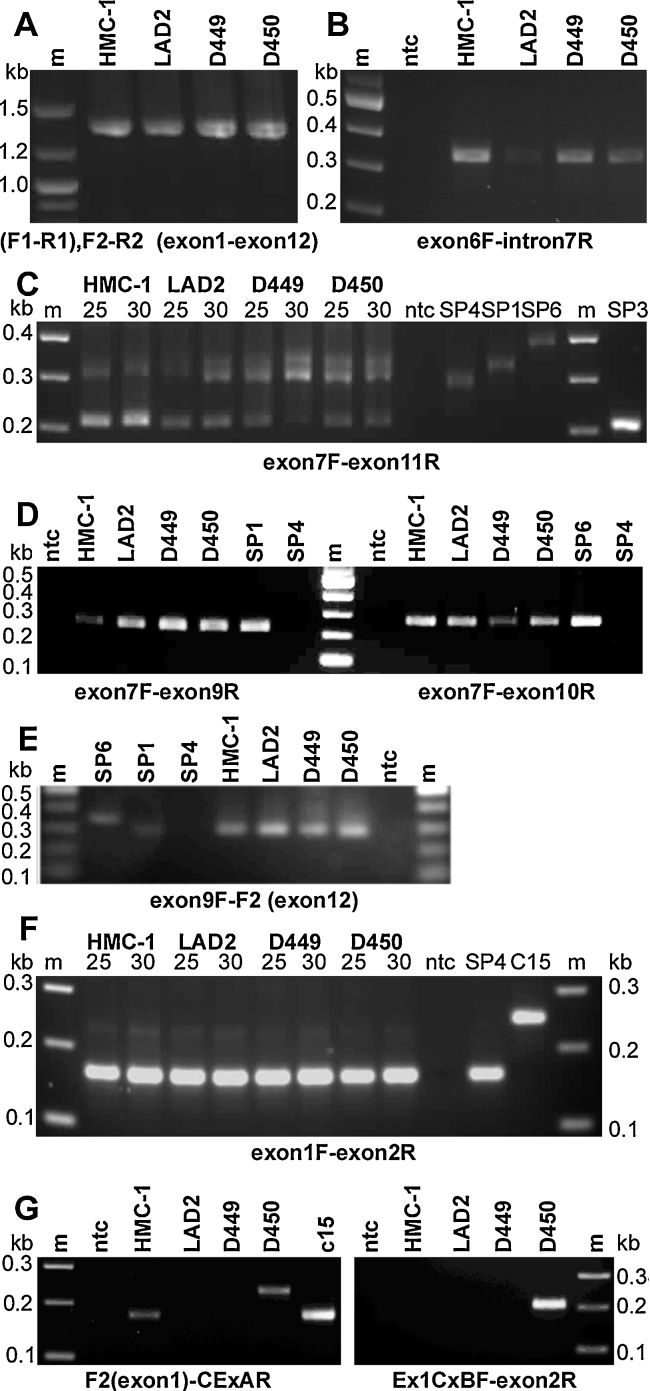
Multiple CADM1 isoforms are expressed in human mast cells. Full-length CADM1 cDNA pools from HLMCs (D449 and D450) and cell lines (HMC-1 and LAD2), obtained by nested RT-PCR are shown in A. Partial cDNAs were amplified by RT-PCR with exon 6/intron 7 primers are shown in B. PCR amplification of full-length cDNA pools (shown above in A) for 25 or 30 cycles with exon-specific primes is shown in C–G. The cDNA fragments were amplified with the primers shown at the bottom of each photograph and separated in agarose gels. Cloned SP4, SP1, SP6 and C15 were used as positive or negative controls in C–G, depending on the primers used in each PCR; non-template controls (ntc) indicates negative control.

**Fig. 2:**
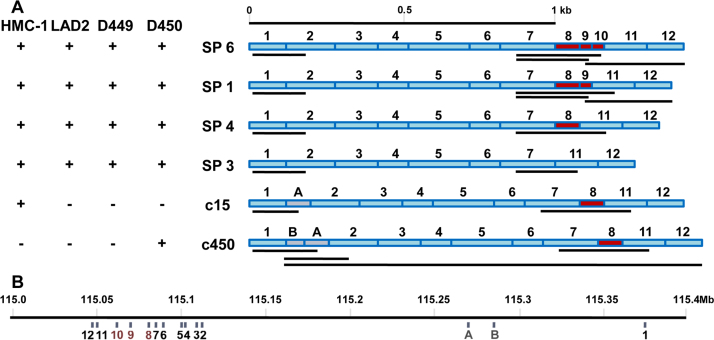
Multiple CADM1 isoforms are present in human mast cells. (A) The diagram summarises CADM isoforms present in HLMCs, D449 and D450, and cell lines, HMC-1 and LAD2. Plus or minus indicates presence or absence of isoforms in the CADM1 PCR pools. The cDNA fragments, amplified for each isoform in Fig. 1C–G, are shown as black lines underneath each isoform. (B) A position of each exon in the human CADM1 gene is illustrated in a diagram, based on information on the CADM1 gene in Ensembl (Flicek et al., 2011). The CADM1 gene is present in an opposite orientation on the chromosome 11q23.

Pools of full-length CADM1 cDNA from HMC-1, LAD2 and HLMCs were analysed for alternative splicing between exons 1 and 2, and exons 7 and 11 using PCR with Pfusion polymerase (New England Biolabs), followed by separation of cDNA fragments in agarose gels. Several alternatively spliced cDNA fragments (shown in Figs. 1 and 2) were purified and sequenced. All sequencing was done by the PNACL at the University of Leicester (http://www.le.ac.uk/mrctox/pnacl/). The sequence of the SP3 fragment has EMBL-EBI ID: HE586501; sequences of c450 fragments have EMBL-EBI ID: HE586502 and HE586503.

In addition, the RNAqueous Micro kit and High Capacity RNA-to-cDNA kit from Applied Biosystems (Life Technologies, UK) were used to produce cDNA from other HLMC specimens and from human normal primary bronchial epithelial cells (gift from Dr Woodman, University of Leicester), obtained from bronchial epithelial samples as previously described (Thomas et al., 2009).

### 2.3 Cryptic exon B analysis

Genomic DNA (gDNA) fragments (390 bp) were amplified, using primers CExBF and CExBR with Pfusion polymerase (New England Biolabs), from gDNA contamination present in total RNA samples isolated with RNA easy kit (Qiagen). Total RNA samples from the MC lines and HLMC specimens were used for amplification of gDNA fragments, which were purified and sequenced on both strands. Similarly, gDNA fragments were amplified from the RNA samples (gift from Latifa Chachi, University of Leicester) from four specimens of healthy airway smooth muscle, isolated as previously described (Kaur et al., 2006). Sequences for gDNA from HMC-1 cells and HLMC D450 have EMBL-EBI ID: HE586622 and HE586621, respectively.

### 2.4 Cell treatments and protein analysis

HLMCs were activated with 2.5 μg/ml IgE (Merck Bioscience) for 40 min, then incubated with 1 μg/ml of anti-IgE mAb (Hybridoma Reagents Laboratory, USA) for 1 h for IgE cross-linking and incubated for 3 h. HMC-1 cells were activated by 100 ng/ml of phorbol-12-myristate-13-acetate (PMA; Calbiochem, Merck Bioscience) for 2 and 3 h. Cells and cell culture medium were collected. Protein extracts were prepared using extraction with triple detergent buffer as previously described (Moiseeva et al., 2012). Cell culture medium was concentrated 10-fold using Amicon centrifugal filters at 4 °C according to the manufacturer's recommendation (Millipore). SDS–PAGE and immunoblotting were performed as previously described (Moiseeva et al., 2012). Blots were probed with anti-CADM1 3E1 IgY mAb (MBL, Japan) followed by anti-Kit mAb (E1, Santa Cruz Biotechnology) and anti-β-actin HRP-conjugated mAb (C4, Santa Cruz Biotechnology), as a whole or in strips.

### 2.5 Quantification of gene expression

All reagents for real-time PCR were purchased from Applied Biosystems (Life Technologies, UK). The cDNA was synthesised using Cell-to-CT kit. Assays-on-demand gene expression kits with Taqman probes and Taqman universal master mix were used to quantify mRNA levels for CADM1, large ribosomal protein P0 (RPL0) and 18S rRNA according to the manufacturer's recommendations. The relative levels of CADM1 expression were calculated by 2^−ΔΔCT^ method (Schmittgen and Livak, 2008), using RPL0 or 18S RNA as a reference, then expressed as a fold change to control.

### 2.6 FACS analysis

Surface CADM1 expression was measured using chicken anti-CADM1 3E1 IgY mAb (Medical & Biological Laboratories, Japan) or IgY control, followed by Cy5-conjugated anti-IgY Ab (Millipore), as previously described (Moiseeva et al., 2012).

### 2.7 Cloning and transfections

Kpn I–Not I-cleaved cDNA fragments of SP4, SP1 and SP6 isolated from clones in plasmid vector pSC-B, described previously (Moiseeva et al., 2012), were recloned into Kpn I–Not I sites of a pcDNA3 plasmid (Invitrogen, Life Technologies, Paisley, UK) for cell expression. The cDNAs of SP4, SP1 and SP6 were re-amplified using oligonucleotides 5′ttcgaattcgacATGGCGAGTGTAGTGCTG and 5′ctcaccggtgAGATGAAGTACTCTTTCTTTTCTTC, cleaved with the restriction enzymes EcoRI and AgeI and cloned in frame into EcoRI–AgeI-cleaved pEGFP-N1 plasmid (Clontech, Takara Bio Europe, Saint-Germain-en-Laye, France) to express CADM1-EGFP-fusion proteins. Plasmids were isolated and sequenced to verify correct PCR amplification and cloning. Cells (10^6^ cells) were transiently transfected with 2.5 μg of plasmid DNA using 7.5 μl of GeneJuice transfection reagent (Novagen, Merck Chemicals, UK) according to the manufacturer's recommendations, and incubated in 10% FBS in DMEM.

### 2.8 Mast cell adhesion

MC adhesion was performed as previously described (Yang et al., 2006) with modifications. LFs or ASMCs were seeded 10^4^ cells/well in 96 well plates in growth medium. When cells reached confluence in 1–2 days, the medium was replaced with DMEM supplemented with insulin/transferrin/sodium selenite (ITS-3; Sigma–Aldrich, UK) for 3 days before use. Transiently transfected HMC-1 cells were labelled with 5 μM calcein AM (Invitrogen, Life Technologies, UK) according to manufacturer's recommendation prior to adhesion. HMC-1 cells (10^4^ cells/100 μl/well) in DMEM adhered to the cell monolayer for 30 min. Then wells were filled with DMEM, plates were sealed and spun upside down at 15 g for 5 min. Medium and non-adherent cells were discarded. Adherent HMC-1 cells were detected by fluorescence.

### 2.9 Data analysis

Statistical analysis was performed using GraphPad Prism 5 software. All data are presented as the mean ± SE. Differences among the groups were analysed using a one-way ANOVA, followed by Dunnett's test to determine whether the groups were different from a control group.

## 3 Results

### 3.1 CADM1 mRNA is alternatively spliced between exons 1/2 and 7/11 in all human mast cells

Previously we synthesised full-length cDNA pools by RT-PCR and cloned CADM1 from the HMC-1 and LAD2 cell lines and two HLMC specimens, D449 and D450 (Moiseeva et al., 2012). Here we used these pools for isoform analysis. They were present as expected bands of ∼1.3–1.4 kb (Fig. 1A). We investigated whether the SP5 isoform was present in human MCs. Using primers as published previously (Koma et al., 2004), we obtained RT/PCR fragments for exon 6–intron 7 (Fig. 1B); however, it was not possible to synthesise a full-length SP5 cDNA (exon 1–intron 7) using various combinations of primers for exon 1 and intron 7, which successfully worked in other RT/PCR reactions (Fig. 1A and B). Hence, the cDNA fragment for exon 6–intron 7 is likely to be an intermediate product of splicing. We also used a RACE protocol to obtain SP5 cDNA, which produced clones encoding various fragments from six chromosomes, but none of them encoded CADM1 (not shown). Hence, we could not demonstrate the presence of the soluble SP5 isoform in human MCs.

Full-length cDNA pools were analysed for alternative splicing. To investigate alternative splicing between exons 7 and 11, CADM1 cDNA pools were amplified with primers exon7F/exon11R. There were three cDNA fragments produced: 292 bp for SP4, 325 bp for SP1 and 208 bp for an unidentified isoform (Fig. 1C and summarised in Fig. 2). Sequencing of the latter fragment identified the SP3 isoform. Interestingly, this isoform was present in all CADM1 cDNA pools and particularly abundant in HMC-1 cells, although none of 109 clones, analysed previously (Moiseeva et al., 2012), represented it. In contrast, the SP4 isoform was less abundant in HMC-1 cells (Fig. 1C), despite the fact that all 25 analysed clones contained exon 8. Hence, the PCR amplification of fragments with exon 8 was less efficient compared to amplification of SP3 fragment. The DNA sequence of exon 8 is unusual: it is comprised of 88% C and A residues, mostly present as a CCA repeat. The SP1 fragment (325 bp) was present in cDNA pools from LAD2 cells and HLMCs, D449 and D450 (Fig. 1C), but not HMC-1 cells, which is consistent with the results from cloning (Moiseeva et al., 2012). The SP6 fragment (379 bp) was not visible in the HLMCs (Fig. 1C), perhaps because it represented only 2–5% of CADM1 clones (Moiseeva et al., 2012).

To examine further the expression of the SP1 and SP6 isoforms in all CADM1 cDNA pools, PCR with specific primers for exons 9 and 10 were used. PCR with primers for exons 7/9 for SP1 and exons 7/10 for SP6 produced single bands 218 bp and 259 bp, respectively, in all cDNA pools (Fig. 1D). Since exon 9 is present in both SP1 and SP6, primers for exons 7/9 amplified the 218 bp fragment from both of them. To distinguish between these isoforms, we used primers for exons 9–12 with all cDNA pools. This set of primers produced 291 bp and 345 bp fragments for SP1 and SP6, respectively. The 291 bp band was amplified from all cDNA pools, whereas the weak 345 bp band was present only in the D449 pool (Fig. 1E), which contained the most of this cloned isoform (Moiseeva et al., 2012). Thus, mRNAs for both SP1 and SP6 were in fact produced in all MCs, but more abundant in differentiated MCs. No other bands were observed, which indicated that neither SP2 (1–7/9–12), nor any other isoforms with exon combinations such as 7/9/11 (CADM1b), 7/10/11 or 7/8/10/11, were expressed in human MCs. All data are summarised in Fig. 2A.

### 3.2 Human mast cells often express mRNA for dysfunctional CADM1 isoforms

Previously we cloned a dysfunctional isoform c15 from HMC-1 cells with an additional cryptic exon A (86 bp) between exon 1 and 2 (Moiseeva et al., 2012). This exon is located in the middle of the intron 1 (Fig. 2B) and shifts the reading frame, inducing premature termination of CADM1 translation. When CADM1 cDNA pools were amplified with exons 1/2 primers, a 164 bp fragment for SP4 was produced (Fig. 1F), but the c15 fragment (250 bp) was not visible in the HMC-1 pool, probably because it represented only 4% of CADM1 clones described previously (Moiseeva et al., 2012). However, using specific exon 1/cryptic exon A primers, we detected a 174 bp c15 fragment in HMC-1 CADM1 pool and another 230 bp fragment in HLMCs from donor D450 (Fig. 1G, left panel). Sequencing of the latter fragment revealed an additional cryptic exon B (56 bp) (Fig. 2). Insertion of this exon also resulted in the coding frame shift and the premature termination of translation. Using a specific primer Ex1CExBF, overlapping the exon 1/cryptic exon B boundary, and an exon 2 primer, we produced a 213 bp fragment only in D450 (Fig. 1G, right panel). Next, the remainder of cryptic CADM1 cDNA, denoted as c450, was obtained using the primers for cryptic exon B (Ex1CExBF) and exon 12 to reveal the presence of exon 8 (Fig. 2A). The expression of cryptic exons was examined further by RT-PCR using primers, as in Fig. 1G, in 3 other HLMC specimens and 3 specimens of primary human bronchial epithelial cells from healthy donors. The cryptic exon A was found in CADM1 cDNA from HLMC D529; whereas the cryptic exon B was not present in any of these samples (not shown). Hence, cryptic exons were found in 3 out of 7 (∼40%) human MC populations analysed.

### 3.3 No specific SNPs are found near or within the cryptic exon B

**Fig. 3:**
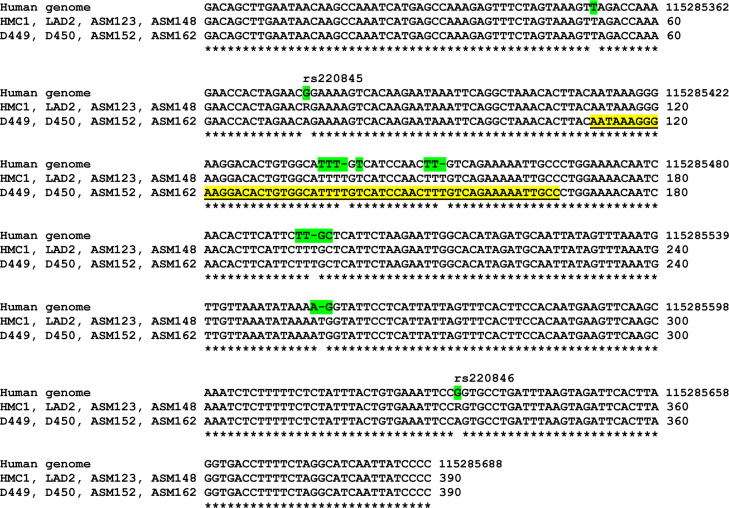
SNPs in and around cryptic exon B are not responsible for its splicing. Genomic DNA fragments from HLMCs (D449 and D450), cell lines (HMC-1 and LAD2) and normal airway smooth muscle (ASM123, ASM148, ASM152 and ASM162) are aligned with the human genome sequence (Ensembl release 68, top strand). A reverse complementary sequence of the cryptic exon B is underlined. Some SNPs are highlighted. Positions of rs220845 and rs220846 are shown as references. R stands for A/G.

Since exon B is located within a hot spot for SNPs (see http://www.ncbi.nlm.nih.gov/SNP/snp_ref.cgi?locusId=23705, some SNPs are shown in Fig. 3), it is possible that specific SNPs within this cryptic exon or its splice branch point (5′ 20–50 bases) may affect CADM1 mRNA splicing. To examine this hypothesis, genomic DNA (390 bp) fragments encompassing the cryptic exon B were synthesised using primers CExBF/CExBR from MC lines, HLMCs and four control specimens of healthy airway smooth muscle cells. Sequencing of 8 DNA fragments identified two groups with either homozygote DNA (D449, D450, ASM153, ASM162) containing adenines in positions 74 (rs220845) and 334 (rs220846) or heterozygote DNA with A/G present in both positions (Fig. 3). Our data are consistent with the reported minor allele frequency for rs220845 (MAF 0.3378) and rs220846 (MAF 0.3434) (Sayers et al., 2011). Although all our gDNA sequences show some mismatch with the genomic sequence (Ensembl release 68), all of them are consistent with SNP polymorphisms reported by NCBI (Sayers et al., 2011). No specific SNPs were found in the cryptic exon B or in close proximity to it in gDNA from HLMC D450; hence, inclusion of this exon was not caused by specific SNPs within this region of the gene.

### 3.4 CADM1 protein in mast cells

**Fig. 4:**
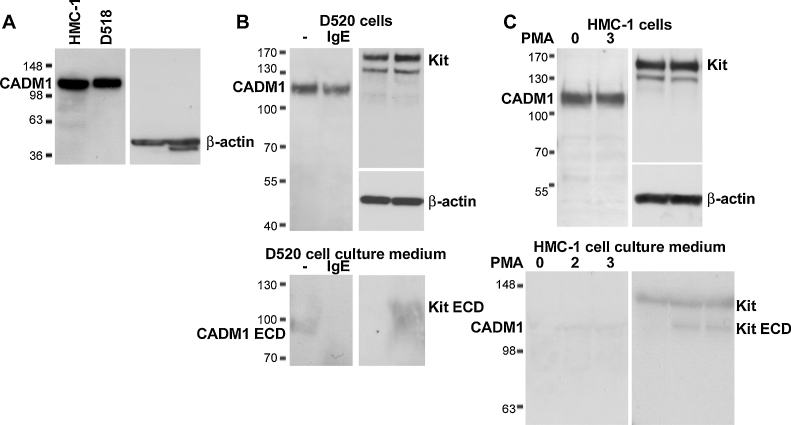
CADM1 protein in HLMCs differs from that in HMC-1 cells. (A) Western blots of proteins from HMC-1 cells and HLMCs from donor D518. (B) Western blots of cell extracts and cell culture medium from HLMCs D520 in the normal state and after 4 h of IgE activation. Representative of experiments with two HLMC specimens. (C) Western blots of cell extracts and cell culture medium from HMC-1 cells in the normal state and after 2 and 3 h of activation with PMA. Representative of two experiments. Cell extracts (30 μg/lane) and cell culture medium (15 μl/lane) were separated in SDS/PAGE, blotted and probed with anti-CADM1 mAb (left side of each panel), followed by anti-Kit and anti-β-actin mAbs (right side of each panel). Positions of CADM1 and Kit extracellular domains (ECD) are shown in B and C.

Since CADM1 is a highly glycosylated protein with a protein core of ∼50 kDa, the isoforms with dual N- and O-glycosylation (∼25 kDa for each type), have very similar molecular weights of ∼105 kDa. CADM1 was present as a single protein band of ∼105 kDa in HLMCs and HMC-1 cells (Fig. 4A–C). HLMC CADM1 from donor D518 had slightly lower mobility in SDS/PAGE than CADM1 in HMC-1 cells (Fig. 4A), which is consistent with our previous results obtained with other 3 HLMCs specimens (Moiseeva et al., 2012). Since HLMCs express ∼20% of SP1 in addition to SP4, and SP1 has slightly higher mobility compared to SP4, the increased weight of CADM1 from D518 may be due to SP1 expression. Several faint bands with mobility ∼55 and 70 kDa, which may represent CADM1 protein core and SP3, could be observed in HMC-1 cells (Fig. 4A and C), particularly at long film exposures.

Since SP1 is reported to be constitutively shed from the cell surface by ADAM17 (Tanabe et al., 2008), we examined if a CADM1 extracellular domain (ECD) could be detected in the MC conditioned medium. A faint CADM1 band ∼95 kDa, equivalent to glycosylated CADM1 ECD, was detectable in cell culture medium in non-activated HLMCs from both donors D520 (Fig. 4B, bottom left panel) and D529 (not shown). However, it was not detectable in the culture supernatant of HMC-1 (Fig. 4C, bottom left panel). Because IgE-dependent activation of MCs upregulates ADAM17 (Sayama et al., 2002), HLMCs were activated by IgE cross-linking. Immature HMC-1 cells, which do not express IgE receptors, were treated with PMA to activate ADAM17 (Cruz et al., 2004). No CADM1 shedding was observed in cell culture media from activated HLMCs or HMC-1 cells (Fig. 4B and C, bottom left panels). No significant changes in CADM1 protein were observed in cell extracts in either case (Fig. 4B and C, top left panels). In contrast, the Kit (CD117) ECD was readily detected in the cell culture medium of IgE-activated HLMCs from donors D520 (Fig. 4B, bottom right panel) and D529 (not shown), and in activated HMC-1 cell culture medium (Fig. 4C, bottom right panel) in agreement with published data (Cruz et al., 2004). We also observed faint bands for full-length CADM1 and Kit in cell culture medium from HMC-1 cells, possibly as a result of cell damage.

Overall these results are consistent with the constitutive shedding of SP1 by HLMCs, and the absence of significant SP1 protein expression in HMC-1, which is in keeping with our previous cloning data (Moiseeva et al., 2012). No any other bands similar to ∼72 kDa SP5 (Koma et al., 2004; Nakahata et al., 2012) were detected in cell culture medium, in keeping with the data obtained by PCR.

### 3.5 Long CADM1 isoforms decreased adhesion of transfected HMC-1 cells to lung fibroblasts but not to airway smooth muscle cells

**Fig. 5:**
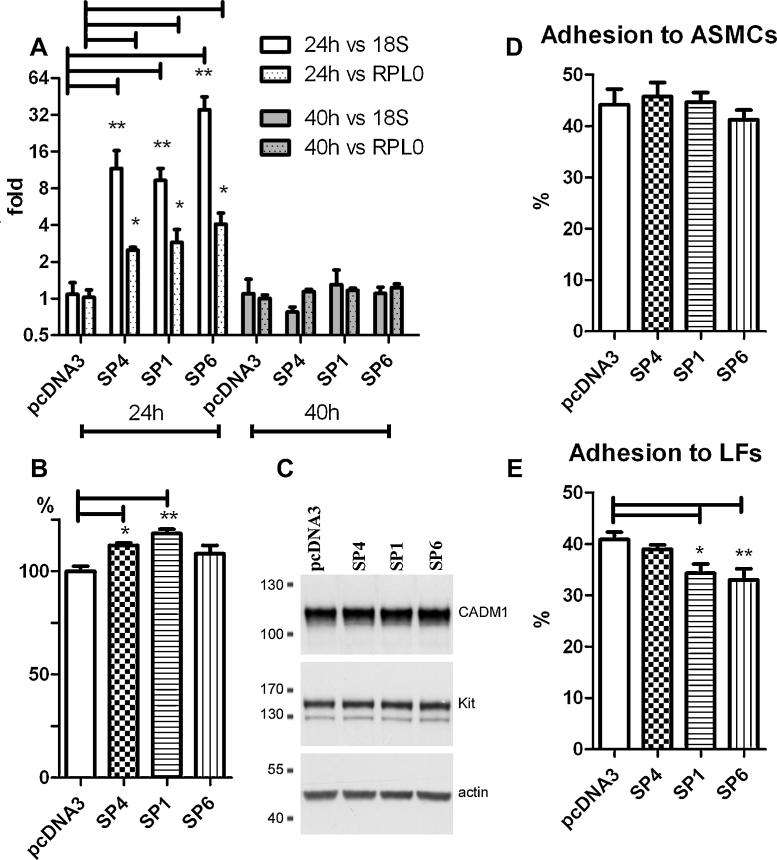
CADM1 isoforms differentially affected HMC-1 cell adhesion to lung fibroblasts, but not to airway smooth muscle cells. Cells were transfected with CADM1 SP4, SP1 or SP6 plasmids or a control pcDNA3 vector. (A) CADM1 mRNA levels are measured 24 or 40 h after transfection and presented as fold increase to control (*n* = 3). (B) Surface CADM1 expression 26 h after transfection is shown as a plot of geometric means (*n* = 3 transfections in duplicate). (C) Immunoblotting of proteins 26 h after transfection. (D and E) Cells transfected for 26 h were studied for adhesion to ASMCs (*n* = 4) in D and LFs (*n* = 5) in E. *P < 0.05; **P < 0.01.

To examine a role of CADM1 isoforms expressed in HLMCs in adhesion, the SP4, SP1 and SP6 isoforms were transfected into HMC-1 cells, expressing predominantly SP4 protein. CADM1 mRNA was significantly increased at 24 h post transfection, when normalised to either 18S rRNA or RPL0 mRNA; and this increase was lost at 40 h (Fig. 5A). Surface and total CADM1 levels were examined at 20, 26 and 30 h post transfection. An increase to 112 ± 1% and 118 ± 2% in surface CADM1 was detected after 26 h in the SP4 and SP1 groups, respectively (Fig. 5B). Transfection with SP6 increased CADM1 amounts slightly to 108 ± 3%. No differences were found after 20 and 30 h (not shown). Western blotting showed similar total CADM1 levels in the SP4, SP1 and SP6 groups compared to control plasmid at the same time (Fig. 5C). Thus, SP4, SP1 and SP6 proteins appeared to be expressed in transiently transfected cells at low levels, despite high mRNA expression. This suggested that CADM1 protein expression in MCs was strictly regulated.

To verify expression of transiently transfected isoforms, SP4, SP1 and SP6 cDNAs were cloned as CADM1–GFP (CADM1 C-terminus fusion) proteins and transfected into HMC-1 cells. Expression of fusion CADM1–GFP proteins was detected in the same transfection conditions (Suppl. Fig. 1). GFP alone was present in the cytoplasm and accumulated in granules inside the cytoplasm (Suppl. Fig. 1). In contrast, green staining for CADM1–GFP fusion proteins was present in cytoplasm with bright speckles at cell outlines for each isoform (Suppl. Fig. 1) in agreement with the reported localisation of SP4 to the cell membrane in MCs (Ito et al., 2003a). No differences between localisation of CADM1 isoforms were found.

**Figure:**
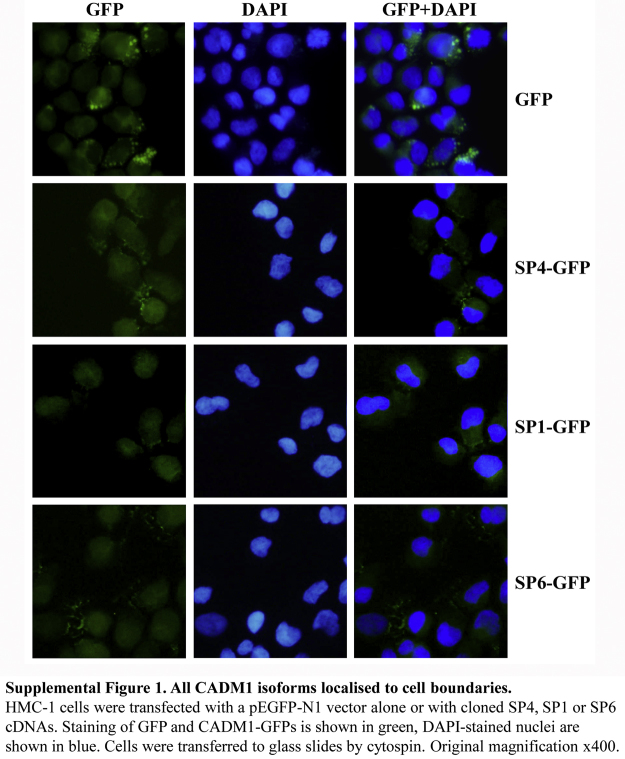

Cells transfected for 26 h were then used in adhesion assays. There were no differences among isoforms in adhesion to ASMCs (Fig. 5D). In contrast, the SP1 and SP6 groups adhered to LFs to a lesser extent (34 ± 2% and 33 ± 2%, respectively) compared to the control pcDNA3 group (41 ± 1%, Fig. 5E). Hence, expression of the longer isoforms on the background of endogenous SP4 decreased HMC-1 cells adhesion to LFs, but not ASMCs.

## 4 Discussion

Analysis of CADM1 at both the mRNA and protein levels, presented here, provides further evidence of marked CADM1 heterogeneity in human MCs. In addition to those isoforms (SP4, SP1, SP6 and c15) cloned recently (Moiseeva et al., 2012), we also found expression of the CADM1 isoform SP3 and a novel cryptic isoform c450. We did not find any other CADM1 isoforms, such as SP2, CADM1b or soluble SP5. The failure to detect SP5 in human MCs is important as it has been cloned from mouse MCs, where it is proposed to function as a negative regulator of CADM1 function (Koma et al., 2004). However, it remains a possibility that the mouse SP5 isoform is in fact an artefact of cloning and not a true isoform. It was cloned as a RT/PCR product generated by oligodT-priming from a short A_22_ sequence in the intron 7, close to the exon 7/intron 7 boundary and present only in the mouse *CADM1* gene; therefore, the mRNA for this clone might have been an intermediate product of CADM1 mRNA splicing. Similarly, the extracellular CADM1 protein, denoted as SP5 previously (Hagiyama et al., 2009; Nakahata et al., 2012), could be a result of proteolytic cleavage of CADM1.

CADM1 therefore undergoes variable splicing between exons 1/2 and 7/11 both in primary human MCs cells and in the human MC lines. Our data suggest that splicing of the *CADM1* gene and its expression in human MCs is far more complicated than that in mouse. Noteworthy, CADM1 is expressed in epithelial cells and neurones to a high extent, but there is no information on expression of CADM1 isoforms in these cells in humans, despite the important non-redundant function of CADM1 in these and other human cells.

Cryptic dysfunctional isoforms were present in ∼40% of MC populations studied, although it must be noted that none of them originated from healthy individuals; all of them were from patients with either lung cancer or MC leukaemia. Cryptic exon A is also present in small partial ESTs BD077366 and DA788329. Cryptic exons may arise as splicing errors in the very long intron 1 (264 kb) or may be caused by specific SNPs, and would occur in various cell types in particular individuals. Our attempt to identify specific SNPs in cryptic exon B or in its splice branch point was not successful. Expression of these dysfunctional isoforms in CADM1-expressing cell types might reduce the CADM1 pool of functional mRNA and, thus, decrease CADM1 proteins levels. Since CADM1 is a tumour suppressor in several cell types (Fukuhara et al., 2002; Ito et al., 2003b; Kuramochi et al., 2001; Nowacki et al., 2008), this may predispose to various cancers and other diseases in which CADM1 is implicated, such as venous thrombosis (Hasstedt et al., 2009; Ito et al., 2003b; Nowacki et al., 2008; Uchino et al., 2003; You et al., 2010). In addition, expression of dysfunctional CADM1 mRNAs in MCs may decrease CADM1 protein levels, downregulating their survival, and attenuating their involvement in pathogen surveillance and their anti-tumourigenic potential in lung cancer (Welsh et al., 2005). Conversely, the presence of these dysfunctional isoforms may protect the same patients against certain MC-dependent diseases, such as asthma and allergy. It is interesting to note that mRNA for the truncated dysfunctional high-affinity IgE receptor FcepsilonRIbeta with an intron sequence is expressed in differentiated human MCs (Cruse et al., 2010). A pro-survival function of CADM1 and functional FcepsilonRIbeta receptor would contribute to persistence of inflammation and allergic reactions. Therefore, it is feasible to consider the existence of dysfunctional mRNAs for both receptors as an evolutionary tool to curb the MC function in inducing inflammation, similar to the loss of functional caspase-12 in humans of Eurasian ancestry in order to reduce sepsis-related lethality (Saleh et al., 2004).

Previously we found by cloning that functional CADM1 in differentiated HLMCs comprises ∼80% SP4, 16–20% of SP1 and 2–5% SP6 (Moiseeva et al., 2012). The clones with a direct and opposite orientations of all CADM1 isoforms did not show differences in sizes of bacterial colonies. Hence, these CADM1 isoforms are not toxic and do not affect bacterial growth. Therefore, the frequency of each isoform among the clones is likely to have reflected its frequency in the CADM1 mRNA populations. Here we examined CADM1 cDNA pools for short specific regions of cDNA using RT-PCR, which is an extremely sensitive method compared to cloning. We found that, when we used primers within regions present in all isoforms (exons 1/2 and exons 7/11), SP3 and SP4 were present in all CADM1 cDNA pools; SP1 was expressed in differentiated MCs, such as LAD2 and HLMCs; whereas the isoforms present in low abundance, such as SP6 or c15, were not amplified in quantities visible in agarose gels. In contrast, SP3 was amplified in CADM1 cDNA pools very efficiently, particularly in HMC-1 cells, but it was not present among 109 CADM1 clones analysed previously (Moiseeva et al., 2012).

However, when we used primers for the specific functional isoforms SP1 and SP6, they were present in all MCs studied. Interestingly, when we used primers for exons 9–12, the SP1 fragment was amplified in all cDNA pools, but the SP6 fragment was present only in the D449 pool. This is consistent with cloning: most of SP6 clones were found in D449 (5% SP6, 16% SP1, 79% SP4), compared to D450 (2% SP6, 18% SP1, 80% SP4), or MC lines LAD2 (20% SP1, 80% SP4) and HMC-1 (4% c15, 96% SP4) (Moiseeva et al., 2012).

Therefore, PCR amplification of cDNA between exons 7/11 favoured the SP3 sequence and was biased against exon 8-containing isoforms, probably due to sequence peculiarity of this exon with CCA repeats. Thus, CADM1 mRNA undergoes extensive splicing in both primary HLMC and neoplastic human MC lines. However, the expression of the longer isoforms is relatively greater in differentiated MCs (HLMCs, LAD2) compared to poorly differentiated MCs (HMC-1). Collectively these PCR results taken together with the efficiency of cloning for CADM1 isoforms imply that the longer SP1 and SP6 isoforms are characteristic of differentiated HLMCs. The mechanisms regulating an abundance of longer CADM1 isoforms in differentiated MCs are not known.

At the protein level, CADM1 showed differences in HLMCs compared to HMC-1 cells. The SP3 isoform (70 kDa) was barely detectable at the protein level (see Fig. 4A and C). CADM1 had low mobility in HLMCs, indicating an abundance of the longer isoforms. SP1 contains a cleavage site in the 11 amino acid sequence encoded by exon 9 (DTTATTEPAVH) and is reported to be constitutively shed by ADAM17 on the basis of using a broad-spectrum metalloproteinase inhibitor TAPI (Tanabe et al., 2008). SP6 contains the same cleavage site. We found evidence of constitutive CADM1 shedding in HLMCs suggesting that substantial amounts of SP1/SP6 protein are present in these cells, but not HMC-1, which is consistent with the PCR results. From a functional perspective, the consequences of CADM1 shedding are not known and require further investigation, and identification of a specific sheddase for CADM1 will require specific inhibitors or other approaches.

Here we investigated adhesion properties of SP4, SP1 and novel SP6, since these isoforms are found in HLMCs. Using transfection we found that despite high CADM1 mRNA levels, protein expression was not markedly increased in transfected cells, suggesting that CADM1 protein levels were regulated. This is in agreement with reported data showing that exogenous CADM1 interferes with the expression of endogenous CADM1 protein (Hagiyama et al., 2011; Moiseeva et al., 2012).

When, SP1 and SP6 are expressed in transfected HMC-1 cells on the background of constitutive SP4, they reduced adhesion to LFs compared to that of the control and SP4-overexpressing cells. No differences were found in adhesion to ASMCs. Previously Hagiyama et al. (2011) have shown that homophilic MC adhesion to neuroblastoma cells has various strength depending on specific CADM1 isoforms expressed in neuroblastoma cells. Therefore, differences in MC adhesion to LFs and ASMCs could be caused by different CADM1 co-receptors expressed in LFs compared to ASMCs. Microarray data in the NCBI record GDS1402 (Sayers et al., 2011), indicate that human LFs and various SMCs express similar mRNA levels of PVRL3 (nectin-3) and CADM3, but different CADM1 levels, which are higher in LFs compared to SMCs. In addition, relatively high CADM1 mRNA expression levels [CADM1 > nectin3 ≫ CADM3, CADM4, CRTAM] are also reported in human embryonic lung fibroblasts in the NCBI record GDS2445 (Bracken et al., 2006). Hence, MC adhesion to LFs and ASMCs is likely to involve several CADM1 counter-receptors, but homophilic CADM1-CADM1 interactions are likely to occur to a higher extent in MC adhesion to LFs rather than to ASMCs. We have found previously that homophilic adhesion is stronger for HMC-1 cells expressing the SP4 isoform compared to the cells expressing mixed SP1/SP4 isoforms (Moiseeva et al., 2012), supporting these data that mixed isoform expression is inefficient with respect to adhesion.

The effect of mixed isoforms on cell adhesion could be explained by receptor dimerisation. Hagiyama et al. (2011) showed that SP4/SP3 or SP4/CADM1b isoforms do not form heterodimers and CADM1 dimers are always homodimers. We proposed a mechanistic model of homophilic MC adhesion to take into account mixed isoforms expressed in human MCs (Moiseeva et al., 2012). Since SP1 and SP6, in particular, have longer extracellular domains, they cannot align and dimerise with the shorter SP4. This would result in slower dimerisation of SP4/SP4, SP1/SP1 and SP6/SP6 in cells expressing mixed isoforms compared to faster dimerisation of SP4/SP4 in cells expressing only this isoform in similar amounts.

In summary, unlike mouse MCs, human MCs express multiple CADM1 isoforms at the mRNA and protein levels. Several dysfunctional isoforms are likely to reduce CADM1 protein levels. Different functional isoforms may differentially affect MC adhesion to some structural lung cells. Further investigations into the mechanisms regulating CADM1 splicing, and the functions of specific CADM1 isoforms are required to evaluate the importance of their expression not only in human MCs, but also in other cell types. Importantly, future studies should include an analysis of CADM1 isoform expression in both health and disease.

## Conflict of interest

The authors declare no competing financial interests. The funders had no role in study design, data collection and analysis, decision to publish, or preparation of the manuscript.

## Figures and Tables

**Table 1:** List of oligonucleotides.

Name	Sequence 5′ → 3′	Location in the *CADM1* gene
F1	ggaggcagccaacgccgcca	5′UTR, exon 1
F2	ggacATGGCGAGTGTAGTGCTG	exon 1, includes initiation codon (ATG)
exon1F	CCGAGCGGATCCCAGTGT	exon 1
exon2R	GTCGCAACCTCTCCCTCGAT	exon 2
CExAR	CCATCCATTTTGAAGCTGATGT	cryptic exon A, intron 1
Ex1CExBF	CTGATCCCCACAGGGCAATTTTTC	exon 1/cryptic exon B boundary
CExBF	gacagcttgaataacaagccaaat	intron 1, 3′ – of cryptic exon B
CExBR	ggggataattgatgcctagaaaag	intron 1, 5′ – of cryptic exon B
exon6F	AGCCTCAAGTGCACATTCAGATGA	exon 6
exon7F	CTGGGCCCAACCTGTTCATC	exon 7
ExIn7R	cttctcacgtacCGTATACATACAG	intron 7/exon 7 boundary
intron7R	aatcacaagtagcagctccatgtg	intron 7
exon9F	ACACAACGGCGACGACAGAACC	exon 9
exon9R	TTCTGTCGTCGCCGTTGTGT	exon 9
exon10R	TCTGCGGAATTGGGCAAC	exon 10
exon11R	AAGCACAGCATGGCGAACAC	exon 11
R1	ggccagttggacacctcattgaa	exon 12, 3′UTR
R2	ggctgatCTAGATGAAGTACTCTTT	exon 12, includes stop codon (TAG)
